# QuinceSet: Dataset of annotated Japanese quince images for object detection

**DOI:** 10.1016/j.dib.2022.108332

**Published:** 2022-05-29

**Authors:** Edīte Kaufmane, Kaspars Sudars, Ivars Namatēvs, Ieva Kalniņa, Jānis Judvaitis, Rihards Balašs, Sarmīte Strautiņa

**Affiliations:** a Institute of Horticulture, Graudu Str. Ceriņi, Krimūnu pag.1, Dobeles nov., LV-3701, Latvia; b Institute of Electronics and Computer Science, Dzērbenes str.14, Riga, LV-1006, Latvia

**Keywords:** Computer vision, Precision Horticulture, *Chaenomeles japonica*, Fruit Detection

## Abstract

With long-term changes in temperature and weather patterns, ecologically adaptable fruit varieties are becoming increasingly important in agriculture. For selection of candidate cultivars in fruit breeding or for yield predictions, fruit set characteristics at different growth stages need to be described and evaluated, which is largely done visually. This is a time-consuming and labor-intensive process that also requires sufficient expert knowledge. The annotated dataset for Japanese quince - QuinceSet - consists of images of Japanese quince (*Chaenomeles japonica*) fruits taken at two phenological developmental stages and annotated for detection and phenotyping. First, after flowering, when the second fruit fall is over and the fruits have reached 30-50% of their final size, and second, at the ripening stage of quince, just before the fruits are yielded. Both stages of quince images classified as unripe and ripe were annotated using ground truth ROI and presented in YOLO format. The dataset contains 1515 high-resolution RGB *.jpg* images with the same number of annotated *.txt* files. Images in the dataset were manually annotated using LabelImg software. A total of 17,171 annotations were provided by the experts. The images were acquired on site at the Institute of Horticulture in Dobele, Latvia. Homogenization of the images was performed under different weather conditions, at different times of the day, and from different capturing angles. The dataset contains both fully visible quinces and quinces partially obscured by leaves. Care was also taken to ensure that the foreground, which contains the leaves has adequate brightness with minimal shadows, while the background is darker. The presented dataset will allow to increase the efficiency of the breeding process and yield estimation, to identify and phenotype quinces more reliably, and may also be useful for breeding other crops.

## Specifications Table

**Table:** 

Subject	Agriculture Engineering, Computer Vision and Pattern Recognition
Specific subject area	Multi-object classification, Object detection, Object recognition, Fruit growth and development
Type of data	Raw data: RGB image(s)Annotations: YOLO format
How data were acquired	Images were captured using Samsung Galaxy A8 camera with ground truth was manually annotated by identifying ROI by LabelImage software.
Data format	Raw images: *.jpg*Annotated data: *.txt* (ROI, groundtruth)
Parameters for data collection	The images were captured at multiple angles in different weather and lighting conditions in an in-field environment.The dataset is composed of 1515 high-resolution RGB images (3456 × 3456 pixels) in .*jpg* format. All 1515 images are accompanied by an annotation that provides ROI (*.txt* format) that provide membership classes for a significant number of pixels.
Description of data collection	The collection of the images was done in-field, at daylight in sunny, when the sun shines on the quince and cloudy weather when the quince is shady. Images are represented from different angles and from the top view to the side view of the fruits. Annotated images were obtained using LabelImg version 1.8.6 software.Dataset Quince_data_set.zip consists of 1515 .*jpg* images and 1515 *.txt* files.
Data source location	Institute of Horticulture, a public experimental facility in Dobele, LatviaCoord WGS84 56°37′335″’ N, 23°33′233″ E
Data accessibility	With the article.https://zenodo.org/record/6402251
Related research article	E. Kaufmane, S. Ruisa, 2020. Breeding of New Cultivars of the Fruit Crop Japanese Quince (Chaenomeles japonica) in Latvia, Acta Horticult. 1281, 51-58. https://doi.org/10.17660/ActaHortic.2020.1281.9

## Value of the Data

• Japanese quince (Chaenomeles japonica) as a fruit plant is a comparatively new crop, so there is relatively little research on it. The more the fruit composition is analyzed, including non-invasive methods, the less cultivation and selection issues are addressed with manual measurements. Therefore, the publicly available dataset for Japanese quince presented here, which includes data from unripe and ripe quince annotated with ground truth ROI for Japanese quince detection and phenotyping, should play a central role in helping breeders develop phenotyping strategies.
• The dataset contains annotated image data classified into two classes according to the phenological stage of development of Japanese quince: unripe and ripe Japanese quince. The first, unripe - about a month after flowering, when the second fruit fall is over underdeveloped fruit sets had already fallen), and yield can be statistically predicted. The second, ripe - when the fruits are fully ripe and the yield can be estimated.
• The precision agriculture community can benefit from these data to detect, evaluate, and monitor the Japanese quince breeding process and test more effective yield prediction more accurately.
• The presented dataset can be used by researchers for image processing pipelines and model calibration in computer vision and for training, testing, and validating Convolutional Neural Networks and Visual Transformers.
• The dataset can be used by researchers to develop and train quince classification and recognition models, and to develop new phenotyping algorithms.
• Farmers can use cell phones in combination with other technological means (e.g., drones) to predict and evaluate the harvest of Japanese quince.

## 1 Data Description

The annotated Japanese quince (*Chaenomeles japonica*) dataset folder contains 1515 original raw images of Japanese quince. Each image was saved in *.jpg* format and have a size of 3456 × 3456 pixels. Each image is accompanied by the same number of *.txt* files in YOLO [1] format annotating the ground truth regions of interest (ROI) of individual Japanese quince.

The YOLO format was chosen for its one-level representative detection architecture in the field of DL to detect plant, locate the region of the plant in the image, and determine the specific category of each object [2]. Compared to two-level models, one-level models are processed faster to detect and count fruits [3]. In the case of quince detection, we chose YOLO because it can be processed quickly and must detect relatively small quinces.

Together with image and annotation files, the *classes.txt* file is included, which contains each class label information. In total, the dataset folder contains 3031 files, but 1515 annotated files comprise 17,171 annotations.

The data contained in the dataset are divided into two classes: (1) the raw images of unripe quinces, the corresponding class value 1 (2) the raw images of ripe quinces, the corresponding class value 0. Images were captured in field conditions: (1) for unripe quinces in June 2021 and for ripe quinces in August 2021. The image data were collected from different Japanese quince genotypes which can be characterized by different forms of shrubs and fruit shapes. The images were captured in an orchard at the Institute of Horticulture (LatHort) in Dobele, Latvia. Experts from LatHort performed the selection for imaging of Japanese quinces, participated in image acquisition and manual annotation, while Institute of Electronics and Computer Science (EDI) provides software and hardware supporting solutions for the dataset

## 2 Experimental Design, Materials and Methods

### 2.1 Background

*Chaenomeles japonica* is a diploid species belonging to the Maloideae, Rosaceae. It is a dwarf shrub originally from central and southern Japan. Japanese quince was brought to Europe already in 1869 and has ever since been appreciated as an ornamental plant because of its showy, long-lasting flowering. [4]. Latvia was one of the first countries in Europa, that in the 1950-ties started the breeding of *Chaenomeles japonica* as a fruit crop for processing. For the last 30 years Japanese quince as a fruit crop is well known not only in the Baltic countries but also in Ukraine, Scandinavia, Germany and Poland. Fruits are an interesting raw material for the food industry because of their nutritional value. It is known that fruit set and yield are strongly dependent on genotype [5]. In Latvia, the breeding of Japanese quince continued in LatHort in the 1990s with the aim of obtaining local cultivars adapted to the Latvian climate. Significant differences were found between different genotypes in terms of productivity, fruit quality, fruit size, biochemical content, and other traits [6]. After evaluation in LatHort, three cultivars 'Rasa', 'Darius' and 'Rondo' were selected and registered in Latvia. These cultivars are very productive (4-8 kg per bush during full crop); fruits are relatively homogeneous, weigh 40-60 g and ripen in early or mid-September [7]. *Chaenomeles japonica* is an example of a complex trait characterized by target populations of the environment, i.e., meteorological conditions and genotypes.

Currently, LatHort has collected rich genetic material of Japanese quince, and breeding is being actively pursued. A number of promising hybrids have been identified and are under detailed consideration for registration of new cultivars. The genotypes differ in shrub shape, yield, winter hardiness, disease resistance, fruit quality characteristics including shape, color, biochemical composition, etc., and fruit ripening time. The Table 1 summarizes some of the most important parameters of the registered varieties and future genotypes.

**Table 1:** Characterization of morphological traits of Japanese quinces.

Geno-type	Yield from bush (g/bush)	Number of fruits in the bush	Average fruit weight (g)	Maximum fruit weight (g)	Part of the seedbed (%)	Hardness (kg/1cm2)	Characteristics of the fruit	Characteristics of the bush
SR1-1	1334.6	35.9	53.4	74.8	8.9	118.4	Round, slightly flattened, smooth with slight ribbing, very deep inflorescence.	Upright shrub, plant habit - vigorous
SR1-2	1717.5	40.6	44.5	63.9	7.0	87.1	Round, smooth, bright yellow, homogeneous, barrel-shaped.	Medium upright shrub, plant habit - moderately vigorous
SR1-3	1514.4	24.7	63.7	88.2	7.2	85.3	Dark yellow round with pronounced red dots and brown dotted rust, slightly ribbed.	Upright shrub, plant habit - vigorous, forms many branches
SR1-4	2067.8	65.1	33.9	53.8	9.8	84.0	Bright yellow, round, barrel-shaped, some even pear-shaped (pyriform), with a very smooth surface, almost without puncture	Upright shrub, plant habit - moderately vigorous
SR1-5	973.9	23.2	41.7	61.6	9.1	103.0	Yellow, round, barrel-shaped, smooth, slightly ribbed at the tip, beautiful fruit, with a few red dots; at the inflorescence a slight brown rust	Medium upright shrub, plant habit - moderately vigorous, bare branches are formed
SR1-6	1544.4	26.9	56.2	74.8	8.5	108.5	Smooth beautiful, round oval or bottle-shaped, slight russeting in the form of small brown dots or stripes.	Upright shrub, plant habit - moderately vigorous
Rasa	1899	55.3	35.5	53	9.4	84.2	Yellow, rounded, a little ribbed, pear-shaped (pyriform) in some years	Semi-erect shrub, branches bend down as a result of high yield in later years;
Darius	874.8	33.0	33.9	44.9	10.1	89.6	Yellow, oblong, smooth, homogeneous	Spreading shrub, plant habit - moderately vigorous
Rondo	1262.2	29.8	42.4	65.0	11.0	90.8	Yellow, oblong, rather homogeneous	Upright shrub, plant habit - vigorous, forms many branches
Ada	1635.2	31.4	52.1	78.2	10.8	81.2	Dark yellow with a pink wreath, oblong, homogeneous	Medium upright shrub, plant habit - moderately vigorous
Alfa	1428.4	27.1	52.7	67.3	10.1	96.2	Yellow, rounded, a little ribbed	Medium upright shrub, plant habit - moderately vigorous

The process of breeding *Chaenomeles japonica* takes 15-20 years from crossing to variety. To select candidate varieties, the characteristics of several thousand seedlings must be described and evaluated, most of which is done visually. This is a time-consuming and labor-intensive process that also requires sufficient manpower. In addition, visual scoring is relatively subjective, and results may vary among different evaluators [8]. Therefore, the utility of new techniques for non-invasive fruit detection and phenotyping to improve yield performance should be evaluated by adopting Machine Learning (ML) techniques, considering cost-benefit and human-centered considerations.

ML and Deep Learning (DL) techniques have shown very promising results in fruit classification and detection problems [9] and yield quality evaluation [10]. A neat and clean image dataset in precision agriculture [11] supplemented with an image labelling tool [12] is the basic requirement to build accurate and robust ML models for the real-time environment. Previous reviews on the task of fruit detection in the field have reinforced the choice of the RGB camera as the detector of choice because it is inexpensive and easy to implement [13].

### 2.2 Image capturing

**Fig. 1:**
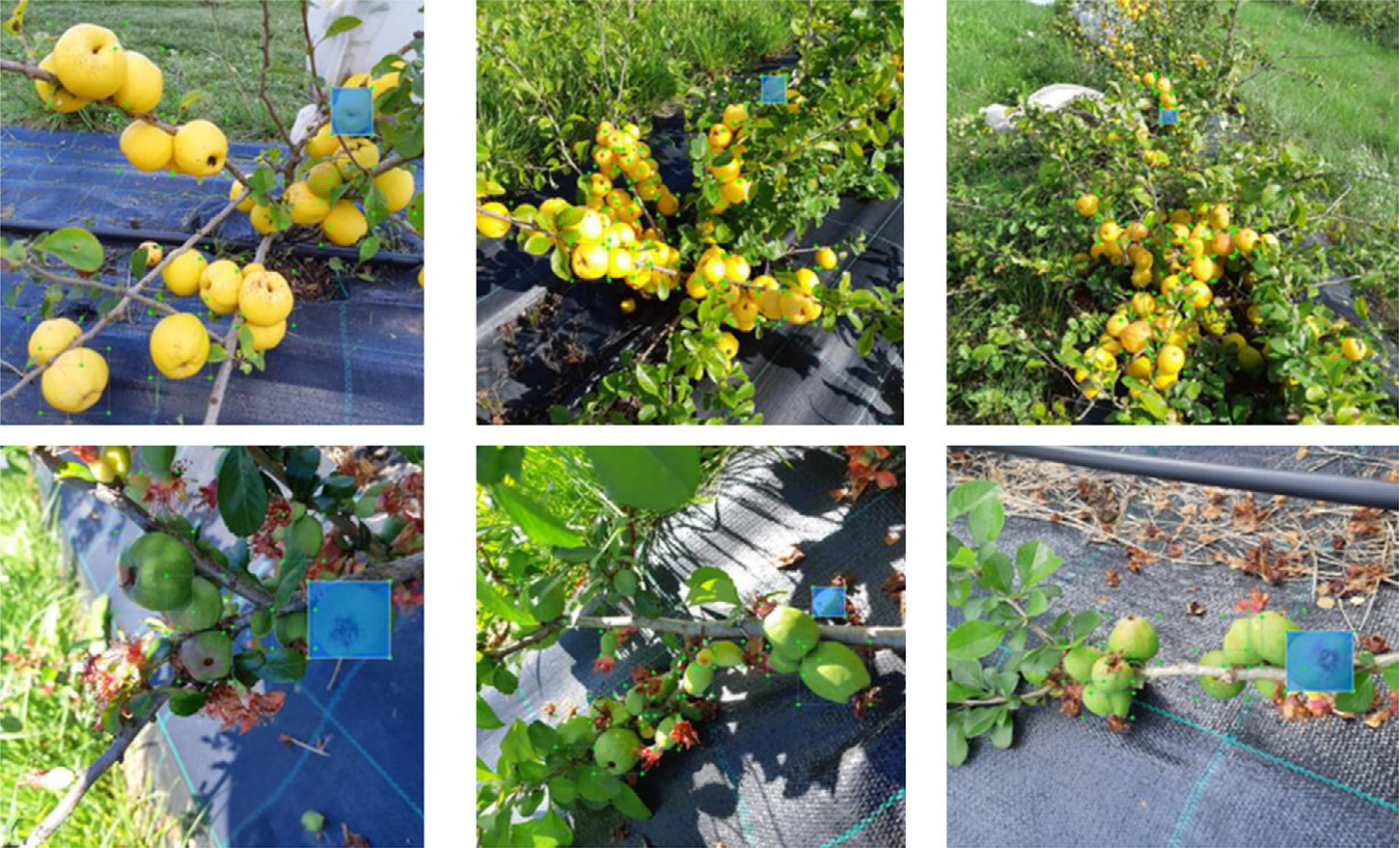
Examples of ground truth labelling of individual Japanese quince using LabelImg software in scenes with varying levels of occlusions by other quinces and leaves. The first row above presents the ripe class images with ROI annotations, the second row the unripe class images with corresponding ROI annotations.

The Japanese quince images were taken in an orchard of the Institute of Horticulture in Dobele, located in the southern part of Latvia (Coord WGS84 56°37′335″ N, 23°33′233″ E). The images were taken on a 0.3 ha plot planted with Japanese quince of eleven genotypes, with an average width of shrub of 0.7-1 m and an average canopy height of 0.5-0.9 m. The images of the Japanese quince were taken with the Samsung Galaxy A8 cell phone, see Fig. 1.

Before image capturing based on the Japanese quince growing stage, experts of the LatHort evaluated its breeding conditions and the best time for imaging The images were captured in a field environment in sunny, cloudy, and partly cloudy weather. The distance between the camera and the Japanese quince for image capture varied from a minimum of 15-20 cm, in which mainly the quinces were seen, to 20-50 cm, in which the quinces and branches were seen, to 50-70 cm, in which the fruits were seen with the shrub and a maximum of 1 m from the plant. In cases where the quinces were not evenly distributed in the shrub, they were captured within distance to capture all quinces. The images were captured from a different angle, including from the top view to the side view of the quinces, and backgrounds.

Capturing of the images took place at two growing phases of Japanese quince cultivation. It means that images were divided into two phenological development stages of quince: (a) unripe and (b) ripe. The first was captured about one month after flowering when the second fruit fall is over (underdeveloped fruit sets had already fallen) and fruits reached 30-50% of final size. The second "portion" was captured at the ripening stage of the quinces, just before the fruits are yielded. Since not all genotypes (cultivars and hybrids) ripen at the same time, three dates were chosen. Data were collected at two different times for unripe quinces and three different times for ripe quinces, see Table 2.

**Table 2:** The time and the metrological conditions during the image collection.

Date	Class	No. of images	Air temperature, °C	Humidity, %	Soil temperature, °C	Soil moisture content, %	PPFD, µmol/m2/s
14.06.2021.	Unripe	449	24.9	35.9	24.0	19.0	1748.6
15.06.2021.	Unripe	440	23.6	45.9	22.9	16.8	1380.8
16.08.2021.	Ripe	46	24.2	57.3	21.5	21.6	958.2
20.08.2021.	Ripe	464	21.3	56.5	19.3	28.9	906.4
23.08.2021.	Ripe	140	22	43.5	20.2	19.7	1205.6

Experts of the LatHort evaluated captured Japanese quince images and divided them into two classes (labels) according to growth stage: (1) unripe and (2) ripe. The images of unripe of Japanese quince were acquired from 14th till 15th June 2021 under daylight. The images of ripe Japanese quinces were acquired on 16th, 20th and 23rd August 2021.

### 2.3 Image annotation

The dataset uploaded to EDI is arranged for a total of 1515 original raw Japanese quince images *.jpg*, which consist of two classes: (a) unripe and (b) ripe images. Label files *.txt* format containing the class names (0, ripe, 1 unripe quince) with associated ground truth ROI boxes were annotated into the format required by YOLO. The dataset contains 17,171 ground truth ROI annotations. The Japanese quince images are annotated using LabelImg version 1.8.6 software [14] with the label name associated with their class. To cover the entire body of quinces using rectangle annotations, some annotations may overlap. The YOLO format stores the annotations in *.txt* file in the following format: object class, object coordinates x, y, height, and width. The values 0 and 1 of the *.txt* file corresponds to the unripe and ripe class, respectively. The following next two values are the x and y coordinates of the annotation, and the rest two are for the height and width of the annotation.

## Ethics Statement

This study did not conduct experiments with humans and animals.

## CRediT Author Statement

**Edīte Kaufmane:** Conceptualization, Methodology, Data Curation, Writing – Review & Editing, Supervision; **Kaspars Sudars:** Methodology, Software, Validation, Data Curation; **Ivars Namatēvs:** Investigation, Writing-Original draft preparation, Visualization; **Ieva Kalniņa:** Investigation; **Jānis Judvaitis:** Software; **Rihards Balašs:** Resources; **Sarmīte Strautiņa:** Project Administration, Funding Acquisition, Methodology.

## Declaration of Competing Interest

The authors declare that they have no known competing financial interests or personal relationships that could have appeared to influence the work reported in this article.

## Data Availability

QuinceSet: Dataset of Annotated Japanese Quince Images for Object Detection (Original data) (Zenodo).
